# Elevated carboxyhaemoglobin as a novel indicator for extracorporeal membrane haemolysis and oxygenator exchange

**DOI:** 10.1186/s13054-021-03582-w

**Published:** 2021-04-27

**Authors:** Kenneth R. Hoffman, Aidan J. C. Burrell, Arne Diehl, Warwick Butt

**Affiliations:** 1 Intensive Care Unit, Alfred Hospital, Melbourne, VIC Australia; 2 Department of Epidemiology and Preventative Medicine, School of Public Health, Monash University, Melbourne, Australia; 3 Intensive Care Unit, The Royal Children’s Hospital, Melbourne, VIC Australia; 4 Faculty of Medicine Nursing and Health Sciences, Monash University, Melbourne, Australia; 5 Paediatric Intensive Care, Murdoch Children’s Research Institute, Melbourne, Australia

Dear Editor,

Plasma free haemoglobin is the gold standard for monitoring for intravascular haemolysis in extracorporeal membrane oxygenation (ECMO), and its use is recommended by the Extracorporeal Life Support Organisation [1]. Elevated plasma free haemoglobin is an independent predictor of mortality during ECMO [2]. Severe haemolysis may herald a hyperfibrinolytic state associated with bleeding, thrombosis and potential membrane oxygenator dysfunction. However, the routine use of plasma free haemoglobin has certain limitations: it is not universally available in all laboratories, it is prone to error due to traumatic sampling, spectrophotometric methods are susceptible to interference from bilirubin and lipaemia and it can take considerable time to perform.

Carboxyhaemoglobin is also used as a marker of intravascular haemolysis. Free haem is degraded by haem oxygenase into biliverdin, free iron and carbon monoxide. This is the primary source of endogenous carbon monoxide production. Several case reports have demonstrated dramatically elevated carboxyhaemoglobin in the setting of haemolysis on ECMO, with no reported cases surviving [3, 4]. There is also evidence of low-level elevation in almost half of veno-venous ECMO patients [5]. The use of carboxyhaemoglobin as a marker of haemolysis has several benefits. It is widely and rapidly available on routinely collected blood gases through co-oximetry, and it is not susceptible to elevation due to traumatic sampling.

In our centre, evidence of haemolysis frequently results in an ECMO circuit exchange. Circuit-driven haemolysis is generally interrupted, normalising the plasma free haemoglobin and preventing progression to a hyperfibrinolytic state or membrane oxygenator dysfunction.

To demonstrate the possible use of carboxyhaemoglobin as a marker of haemolysis on ECMO, we analysed the blood results of four patients on veno-venous ECMO for severe respiratory failure who had circuit exchanges due to suspected intravascular haemolysis with elevated plasma free haemoglobin. Plasma free haemoglobin and carboxyhaemoglobin data were collected for the 72 h before and after ECMO circuit exchange and are presented in Fig. 1.

**Fig. 1:**
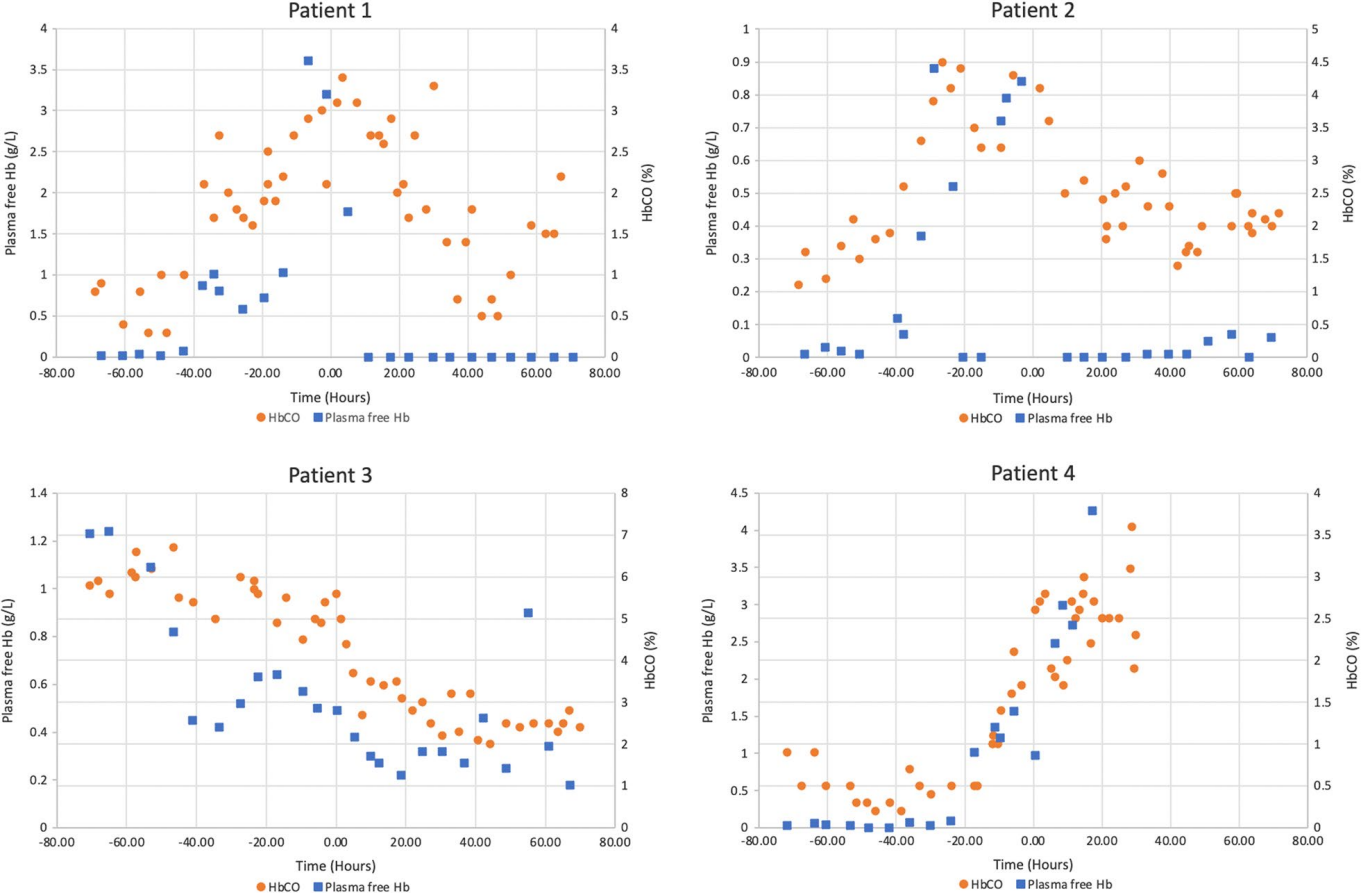
Comparison of carboxyhaemoglobin and plasma free haemoglobin values in the 72 h before and after circuit exchange (time point 0 represents the time of circuit change)

The results demonstrate that carboxyhaemoglobin levels were elevated prior to the circuit exchange in all cases and correlated with acute rises in plasma free haemoglobin. In patients 1–3, carboxyhaemoglobin levels then fell over the subsequent days post-circuit exchange. Patient 4 showed the same correlation, however died of suspected overwhelming sepsis with diffuse intravascular coagulation 30 h post-circuit exchange.

These preliminary results suggest carboxyhaemoglobin is potentially a novel marker of haemolysis on ECMO and may be useful as an indicator for ECMO circuit exchange. Further well-designed prospective studies are needed to investigate whether the relationship between carboxyhaemoglobin and intravascular haemolysis during ECMO is more widespread, and whether it has a clinically useful role in the management of ECMO patients.

## Data Availability

Original data are available on request.
